# Identifying circulating biomarkers for major depressive disorder

**DOI:** 10.3389/fpsyt.2023.1230246

**Published:** 2023-08-04

**Authors:** En Zhang, Zhongfei Huang, Zongjun Zang, Xin Qiao, Jiaxin Yan, Xuefei Shao

**Affiliations:** ^1^Department of Psychiatry, The Fourth People’s Hospital of Wuhu City, Wuhu, China; ^2^College of Humanities and Management, Wannan Medical College, Wuhu, China; ^3^Department of Neurosurgery, The First Affiliated Hospital of Wannan Medical College, Wuhu, China

**Keywords:** major depressive disorder, inflammation, serum, biomarker, characteristic proteins

## Abstract

**Objective:**

To date, the current diagnosis of major depressive disorder (MDD) still depends on clinical symptomatologic criteria, misdiagnosis and ineffective treatment are common. The study aimed to explore circulating biomarkers for MDD diagnosis.

**Methods:**

A high-throughput antibody array technology was utilized to detect 440 circulating cytokines in eight MDD patients and eight age–and gender-matched healthy controls. LASSO regression was conducted for MDD-related characteristic proteins selection. Enzyme-linked immunosorbent assay (ELISA) was used to validate the characteristic proteins in 40 MDD patients and 40 healthy controls. Receiver operating characteristic (ROC) curve was employed to evaluate the diagnostic values of characteristic proteins for discriminating MDD patients from healthy controls. Correlations between the levels of characteristic proteins and depression severity (HAMD-17 scores) were evaluated using linear regression.

**Results:**

The levels of 59 proteins were found aberrant in MDD patients compared with healthy controls. LASSO regression found six MDD-related characteristic proteins including insulin, CD40L, CD155, Lipocalin-2, HGF and LIGHT. ROC curve analysis showed that the area under curve (AUC) values of six characteristic proteins were more than 0.85 in discriminating patients with MDD from healthy controls. Furthermore, significant relationship was found between the levels of insulin, CD155, Lipocalin-2, HGF, LIGHT and HAMD-17 scores in MDD group.

**Conclusion:**

These results suggested that six characteristic proteins screened from 59 proteins differential in MDD may hold promise as diagnostic biomarkers in discriminating patients with MDD. Among six characteristic proteins, insulin, CD155, Lipocalin-2, HGF and LIGHT might be useful to estimate the severity of depressive symptoms.

## Introduction

Major depressive disorder (MDD) is a prevalent psychiatric disorder characterized by symptoms such as depressed mood lasting more than 2 weeks and decreased energy, poor attention, functional impairment, disturbed appetite and sleep, anhedonia and suicide (1). MDD can cause significant disability, and a burden to patients, their families and society. In clinical diagnosis of MDD, brain-imaging techniques such as functional magnetic resonance imaging, electroencephalography, positron emission tomography, and magnetoencephalography are used as adjuncts. However, these clinical examinations are expensive. In addition, International Statistical Classification of Diseases and Related Health Problems-10th revision (ICD-10) or the Diagnostic and Statistical Manual of Mental Disorders, 5th Edition (DSM-5) classifications are also used to diagnose MDD. However, the accuracy remains controversial. An accurate diagnosis of MDD may help to prevent the risk of developing chronicity of depressive symptoms and physical and neuropathological complications such as cerebrovascular diseases (2). Therefore, an effective candidate that can serve as a definite diagnostic marker for MDD is required.

So far, the search for suitable biomarkers for MDD is still prioritized in the biological research. Biomarkers come in various forms, such as inflammatory cytokines, endogenously produced hormones and brain imaging. Studies indicate that long-term stress is associated with increased chronic inflammatory processes, stress and depression can cause immune system disorder, increased leukocyte function, and increased proinflammatory cytokines (3, 4). For example, MDD patients have high levels of proinflammatory mediators such as IL-1, IL-6 and TNF-α, which stimulate the hypothalamic–pituitary–adrenal (HPA) axis to increase the release of glucocorticoids (5). Abnormal cytokines involve into the mechanisms of neurotransmission and neuronal signaling in brain regions associated with MDD. Therefore, a search of cytokines associated with MDD may help to improve the accurate of diagnosis for MDD.

## Materials and methods

### Subjects

This study was conducted at The Fourth People’s Hospital of Wuhu City. Patients diagnosed as MDD according to the Diagnostic and Statistical Manual of Mental Disorders, 4th Edition (DSM-IV) criteria were enrolled into the study. The inclusion criteria were as follows: (1) aged 18–60 years old; (2) met the diagnostic criteria of MDD in DSM-IV; (3) the 17-item Hamilton Depression Rating Scale (HAMD-17) >17; (4) no history of antidepressants, psychotropic drugs and electroconvulsive therapy (MECT) in the past 3 months. The exclusion criteria were as follows: (1) patients with organic brain diseases, other major psychiatric disorders or neurodegenerative illness history; (2) patients with secondary depression induced by physical disease, drug or other mental diseases; (3) patients with serious heart, brain, liver, kidney, immune disorders, obesity, poor nutrition, acute and chronic infection. (4) pregnant women. Health controls were recruited with HAMD-17 score of <7 in the present study. This procedure was approved by the ethics committee of The Fourth People’s Hospital of Wuhu City [(2020)-KY-18], and informed consent was obtained from all the participants.

### Antibody array assay

Peripheral blood from the subjects were collected by venipuncture, and immediately centrifuged at 1, 200 x g for 10 min at 4°C. The supernatant was extracted as a serum, stored at −80°C until determination. Serum samples from 8 patients and 8 healthy subjects were measured with Human Cytokine Antibody Array (GSH-CAA-440, RayBiotech Company, Norcross, GA, United States) simultaneously detecting 440 cytokines according to the manufacturer’s instructions. Briefly, serum samples were added into the array pools to incubate with precoated capture antibodies overnight. After washing, a biotin-conjugated anti-cytokine antibody mix was added into the pools for further incubation for 2 h at room temperature. Finally, after the incubation of Cy3-conjugated streptavidin, the fluorescent signal was measured using an InnoScan 300 Microarray Scanner (Innopsys, France).

### Enzyme-linked immunosorbent assay performance

ELISA kits (RayBiotech, Norcross GA, United States) were performed to validate the antibody array result according to the manufacturer’s instructions with 40 MDD patients, 40 healthy controls. Briefly, after diluted at different dilution factors based on individual serum biomarkers, serum samples were incubated in the plate wells overnight. The next day, after washing with wash buffer, the plate wells were incubated with biotin-conjugated antibody for 2 h. HRP-conjugated streptavidin was added to catalyze following tetramethyl benzidine (TMB) reagent. For each incubation, each well was added 100 μL. Finally, the optical density values were determined at 450 nm using a microplate reader (ELx800NB, Biotek, Winooski, CT, United States).

### Statistical analysis

Comparisons between groups were performed by moderated T test (limma data package, R/Bioconductor) software. Differences were considered statistically significant when *p* values were < 0.05. All data were shown as mean ± SD. Correlation analysis was performed using linear regression.

## Results

### Differential proteins in MDD

**Figure 1:**
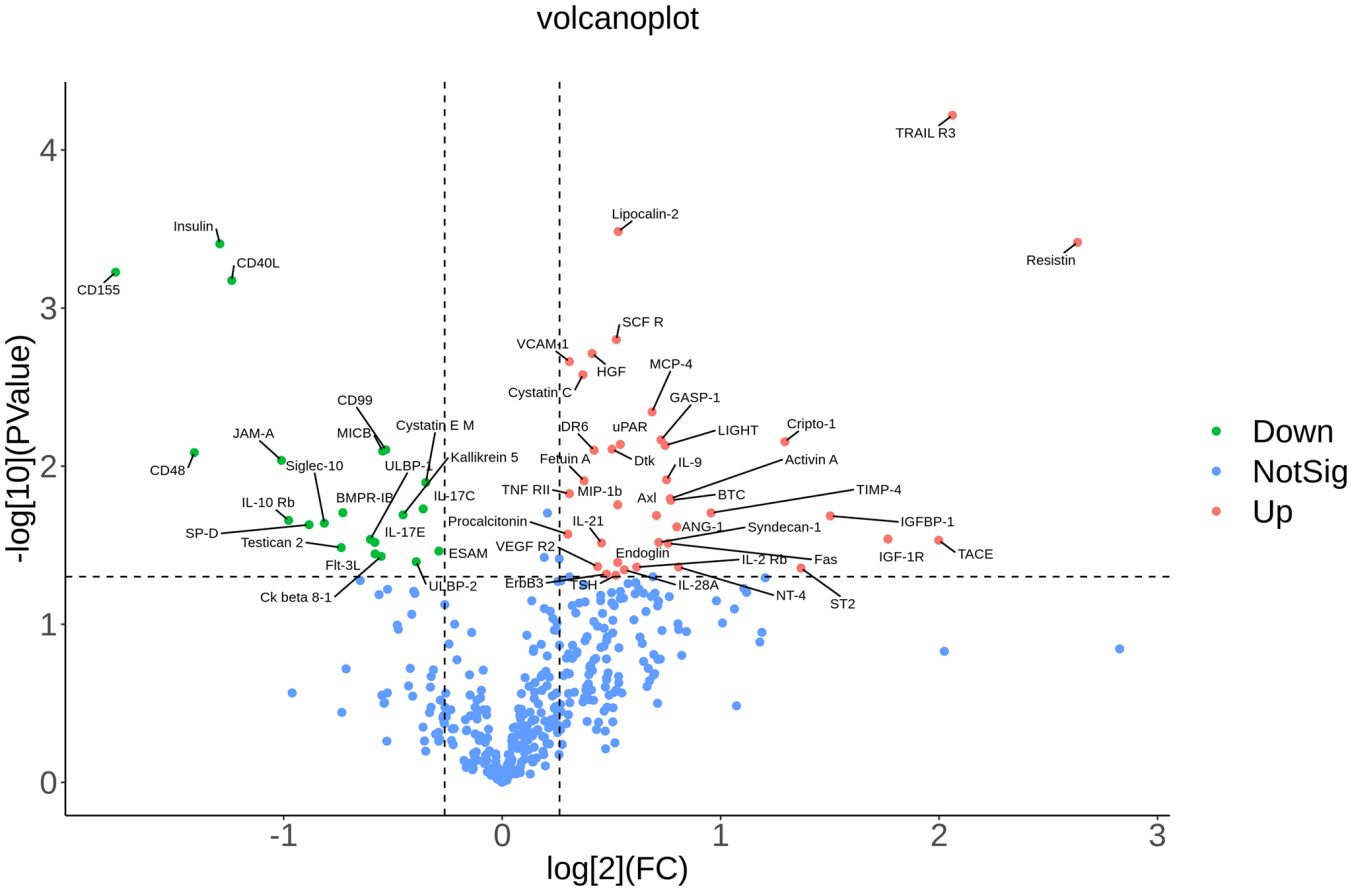
Volcano plot. The differences of 440 cytokines between MDD and healthy controls are shown by fold change (FC, MDD vs. control) and *p* value. Blue dots represent not significant cytokines with *p* value >0.05 and 1.2 > fold change >0.83. Red dots denote upregulated cytokines in MDD with *p* value <0.05 and fold change >1.2. Green dots indicate downregulated cytokines in MDD with *p* value <0.05 and fold change <0.83.

**Figure 2:**
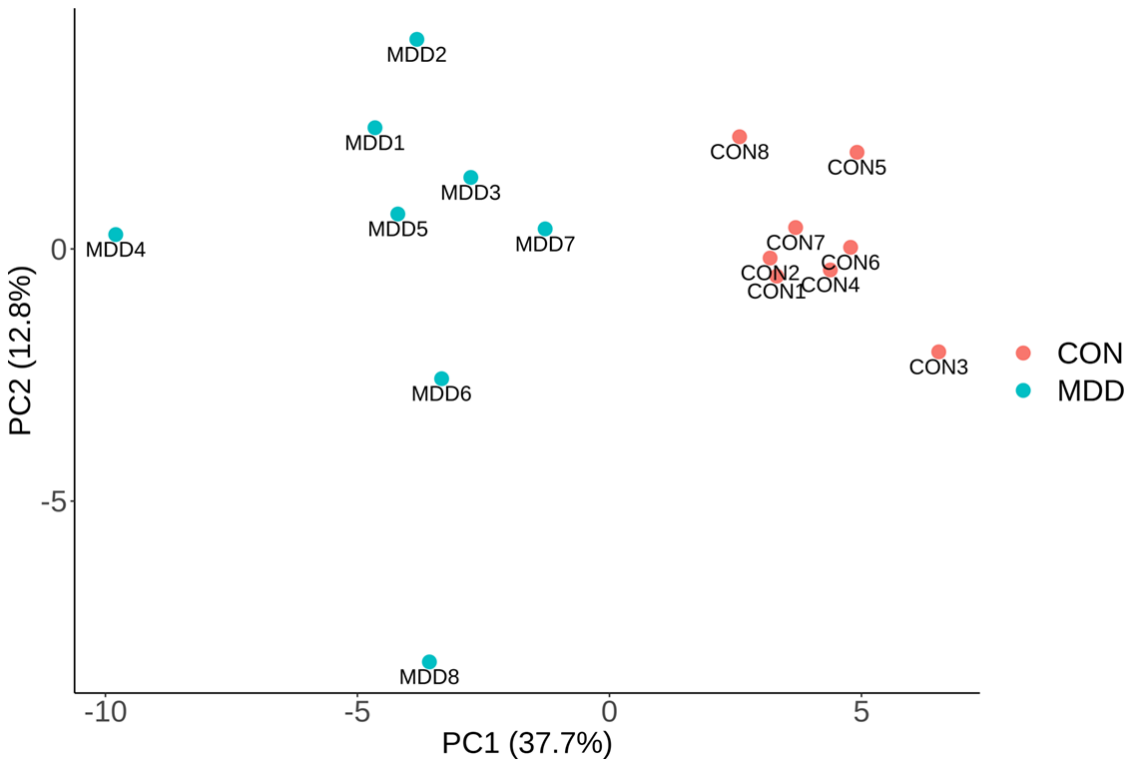
PCA. The differences of 59 significantly altered proteins were presented via PCA using array data. The first two principal components are plotted, and MDD group (blue dots) and control group (red dots) distribute in distant positions.

The data of 440 proteins from antibody array was analyzed to look for differential proteins in MDD. As a result, 59 proteins were found significantly differential in MDD compared with healthy group (Figure 1; Table 1). To validate the difference of these proteins, a principal component analysis (PCA) showed the samples of the MDD group and healthy group located in different positions (Figure 2), indicating the significant differences in these proteins between MDD and healthy groups.

**Table 1:** Fifty-nine differential proteins in MDD as compared with healthy controls.

ProteinID	EntrezID	UniprotID	MDD	Control	*p* value	Foldchange
Insulin	3,630	P01308	2086.50	5505.36	0.000	0.38
CD40L	959	P29965	1250.96	3220.82	0.001	0.39
CD155	5,817	P15151	1584.79	4926.43	0.001	0.32
MICB	4,277	Q29980	574.84	875.81	0.008	0.66
CD48	962	P09326	530.34	1207.72	0.008	0.44
CD99	4,267	P14209	21605.62	29850.75	0.008	0.72
JAM-A	50,848	Q9Y624	380.18	711.39	0.009	0.53
Cystatin E M	1,474	Q15828	49399.61	61911.46	0.013	0.80
IL-17C	27,189	Q9P0M4	1321.31	1726.21	0.019	0.77
BMPR-IB	658	O00238	680.43	1047.46	0.020	0.65
Kallikrein 5	25,818	Q9Y337	2496.59	3454.37	0.020	0.72
IL-10 Rb	3,588	Q08334	568.91	1278.84	0.022	0.44
Siglec-10	89,790	Q96LC7	471.48	767.72	0.023	0.61
SP-D	66,421	P35247	1702.43	3020.84	0.023	0.56
IL-17E	64,806	Q9H293	1120.38	1769.76	0.030	0.63
ULBP-1	80,329	Q9BZM6	383.49	598.78	0.029	0.64
Testican 2	9,806	Q92563	846.44	1401.22	0.033	0.60
ESAM	90,952	Q96AP7	58013.40	69841.54	0.034	0.83
Flt-3 L	2,322	P36888	5693.30	8034.82	0.036	0.71
Ck beta 8–1	6,368	P55773	710.20	1095.73	0.037	0.65
ULBP-2	80,328	Q9BZM5	5348.16	6981.56	0.040	0.77
TRAIL R3	8,794	O14798	30928.79	8678.80	0.000	3.56
Lipocalin-2	3,934	P80188	146588.12	103134.96	0.000	1.42
Resistin	56,729	Q9HD89	21659.21	2984.17	0.000	7.26
SCF R	3,815	P10721	105281.34	74289.45	0.002	1.42
VCAM-1	7,412	P19320	176302.21	142736.03	0.002	1.24
HGF	3,082	P14210	1315.89	991.98	0.002	1.33
Cystatin C	1,471	P01034	117671.80	91706.23	0.003	1.28
DR6	27,242	O75509	159579.99	118642.61	0.008	1.35
Dtk	7,301	Q06418	4259.09	3044.14	0.008	1.40
uPAR	5,329	Q03405	3313.87	2219.61	0.007	1.49
LIGHT/TNFSF14	8,740	O43557	784.82	459.86	0.007	1.71
MCP-4/CCL13	6,357	Q99616	558.92	350.03	0.005	1.60
Cripto-1	6,997	P13385	7686.05	3199.07	0.007	2.40
GASP-1	124,857	Q8TEU8	3290.89	1941.82	0.007	1.69
IL-9	3,578	P15248	750.22	406.92	0.012	1.84
Fetuin A	197	P02765	23712.36	18722.56	0.012	1.27
MIP-1b/CCL4	6,351	P13236	13272.53	9391.81	0.018	1.41
TNF RII	7,133	P20333	103462.81	83288.88	0.015	1.24
IGFBP-1	3,484	P08833	7476.11	2025.39	0.021	3.69
Axl	558	P30530	1150.87	666.97	0.021	1.73
BTC	685	P35070	1576.23	836.60	0.016	1.88
Activin A	3,624	P08476	4035.15	2091.20	0.016	1.93
TIMP-4	7,079	Q99727	23516.75	13162.03	0.020	1.79
ANG-1	284	Q15389	6668.63	3186.72	0.024	2.09
Fas	355	P25445	15996.64	11271.64	0.031	1.42
IGF-1R	3,480	P08069	1467.50	205.21	0.029	7.15
IL-21	59,067	Q9HBE4	1030.01	711.76	0.031	1.45
Procalcitonin	796	P06881	1211.68	997.99	0.027	1.21
TACE	6,868	P78536	2023.02	244.50	0.029	8.27
Syndecan-1	6,382	P18827	2937.75	1675.43	0.030	1.75
Endoglin	2022	P17813	7561.26	5139.89	0.041	1.47
NT-4	4,909	P34130	1104.84	527.83	0.043	2.09
VEGF R2	3,791	P35968	10142.85	7199.59	0.043	1.41
IL-2 Rb	3,560	P14784	2765.70	1592.14	0.044	1.74
ST2	9,173	Q01638	3503.65	885.62	0.044	3.96
IL-28A	282,616	Q8IZJ0	525.25	354.92	0.045	1.48
ErbB3	2065	P21860	16701.41	11560.21	0.048	1.44
TSH	7,252	P01222	1158.15	740.32	0.049	1.56

### Bioinformatics analysis

**Figure 3:**
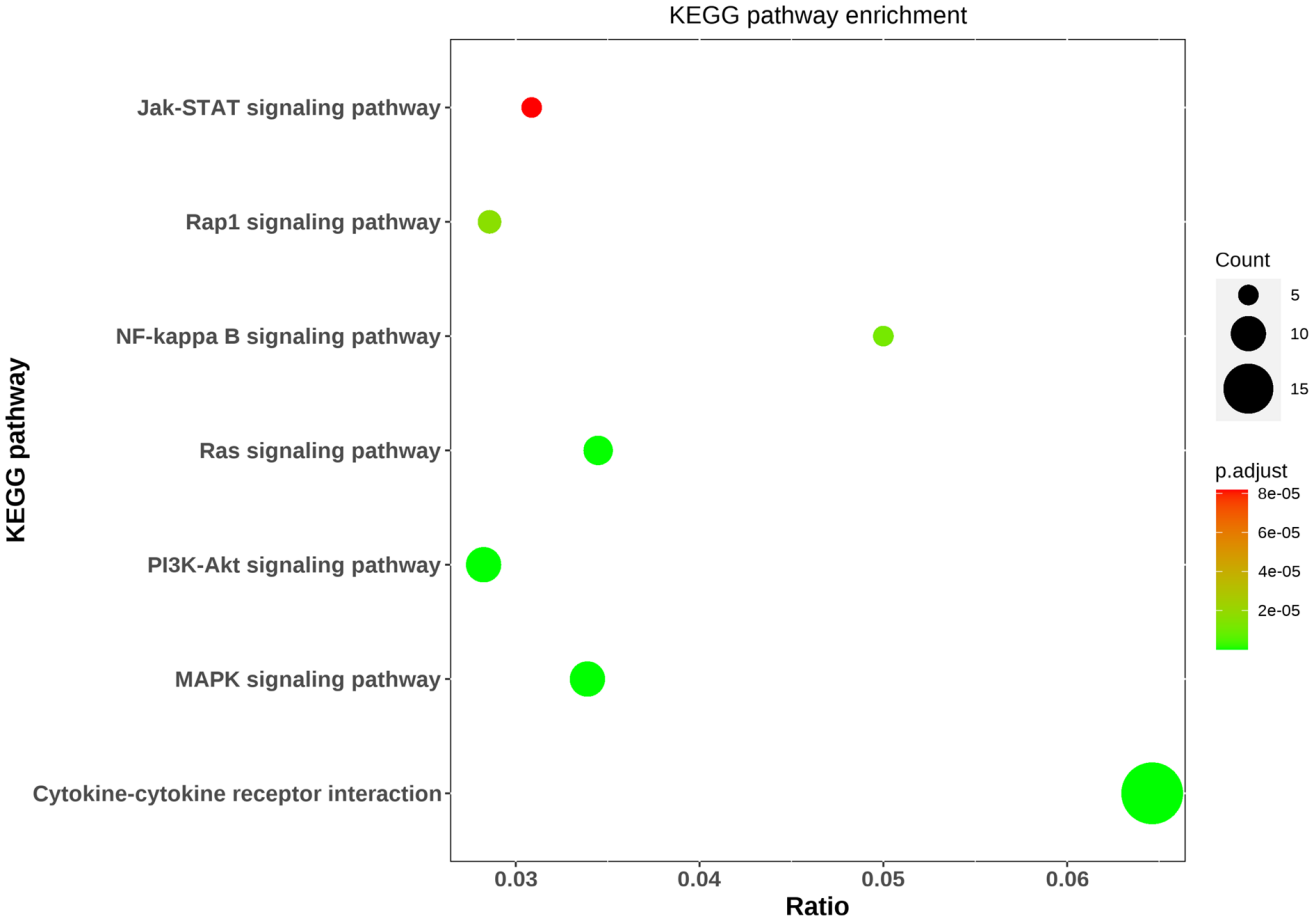
Bioinformatic analysis of differential proteins. The top seven KEGG pathway enrichments were exhibited via bubble diagram. Using Fisher’s accurate test, *p* < 0.05 was considered statistically significant. Count means the differential protein number involving into the KEGG pathway.

Pathway analysis was performed to determine which known pathway network was enriched among these differential proteins. As a result, pathway analysis generated 15 networks from these 59 differential proteins, with 7 networks involving into inflammatory response including the signaling pathways of MAPK, PI3K-Akt, Ras, NFKB, Rap1, JAK–STAT and cytokine-cytokine receptor interaction (Figure 3). And gene ontology analysis showed some differential proteins were enriched in inflammatory response.

### Characteristic proteins selection

**Figure 4:**
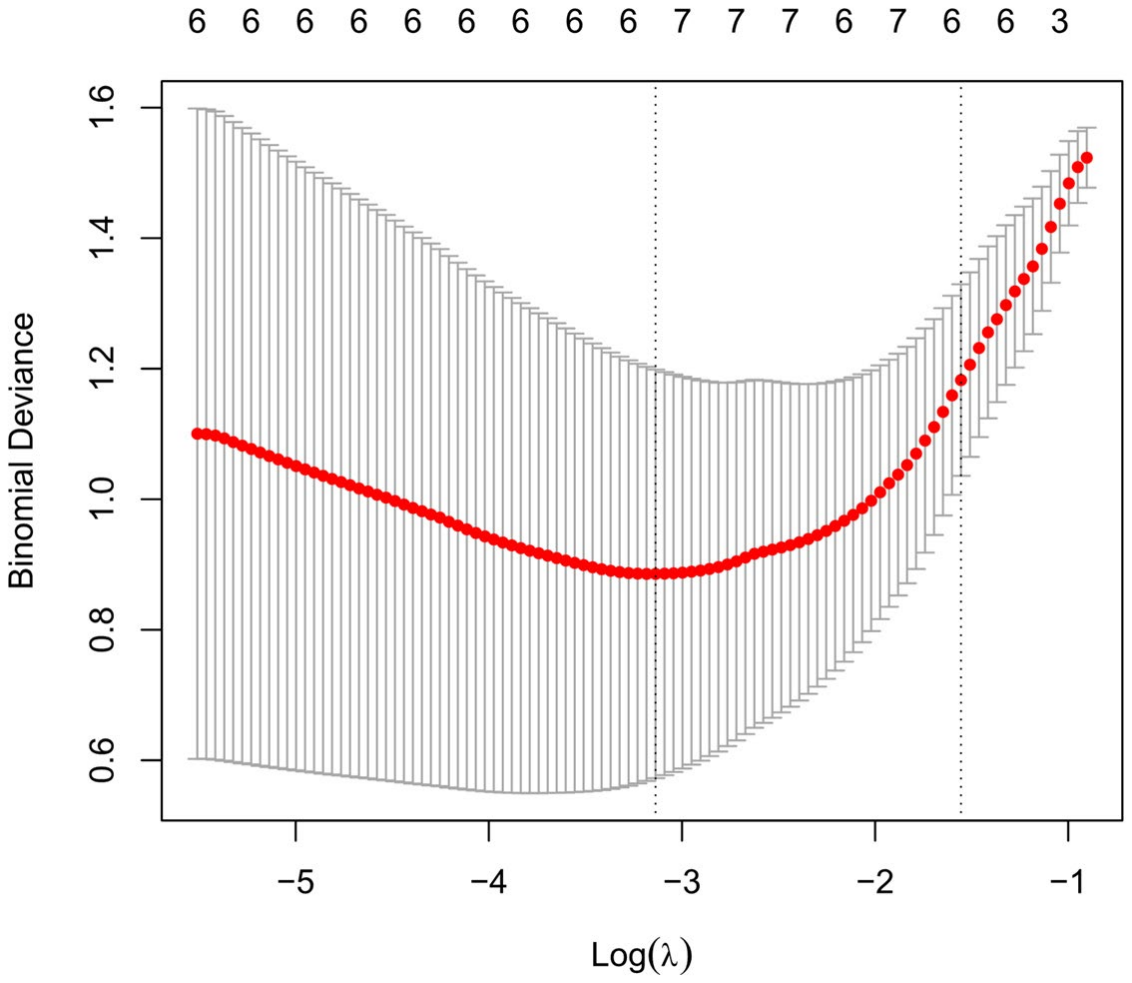
Selection of MDD-related characteristic proteins. LASSO coefficient profiling was performed, a solid hammer line represents binomial deviance and the left dotted line indicates the optimal lambda value.

**Figure 5:**
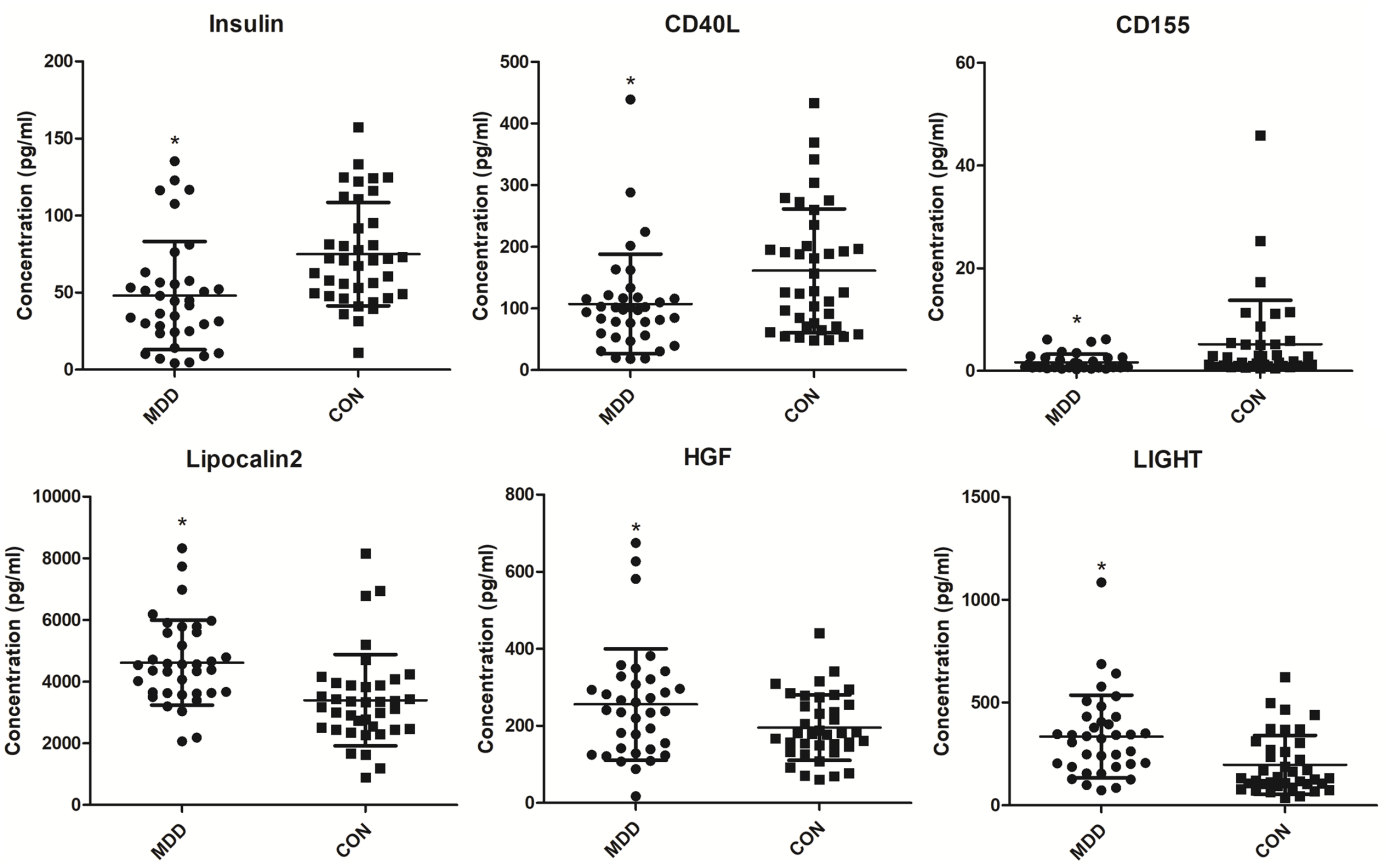
Serum levels of MDD-related characteristic proteins in MDD patients and healthy controls. Serum levels of MDD-related characteristic proteins were determined with ELISA. *p < 0.05 vs. CON. MDD, major depressive disorder. CON, healthy controls.

In order to find MDD-related characteristic proteins among 59 differential proteins, LASSO regression was conducted for model selection. Basing on the optimal lambda of the LASSO regression, six characteristic proteins were found including insulin, CD40L, CD155, Lipocalin-2, HGF and LIGHT (Figure 4). Furthermore, ELISA validation showed identical result to that in antibody array (Figure 5).

### Diagnostic values of MDD-related characteristic proteins

**Figure 6:**
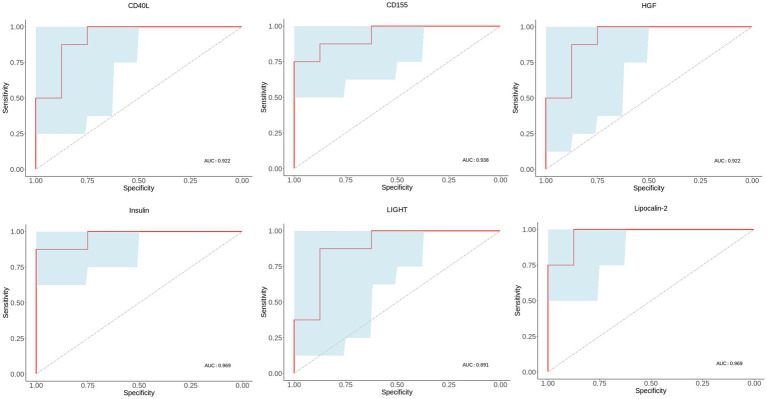
ROC curves of MDD-related characteristic proteins. Blue area in ROC curve represents confidence interval. ROC, receiver operating characteristic. AUC, area under the receiver operating characteristic curve.

To validate the importance of six characteristic proteins screened by LASSO regression in predicting and diagnosing MDD, receiver operating characteristic (ROC) analysis was performed. The ROC curves are shown in Figure 6, and area under the curve (AUC), sensitivity, specificity and 95% confidence interval are exhibited in Table 2. Combined these results, these characteristic proteins exhibited a good predictive and diagnostic performance for MDD.

**Table 2:** ROC analysis of characteristic proteins in discriminating MDD patients from healthy controls.

Protein	Sensitivity%	Specificity%	AUC	95% CI	*p* value
Insulin	87.5	100	0.9688	0.8918–1.046	0.0016
CD40L	100	75	0.922	0.7844–1.059	0.0046
CD155	75	100	0.9375	0.8219–1.053	0.0033
Lipocalin-2	100	87.5	0.9688	0.8918–1.046	0.0016
HGF	100	75	0.9219	0.7844–1.059	0.0046
LIGHT	87.5	87.5	0.8906	0.7214–1.060	0.0087

### Correlation between HAMD-17 scores and characteristic proteins

**Figure 7:**
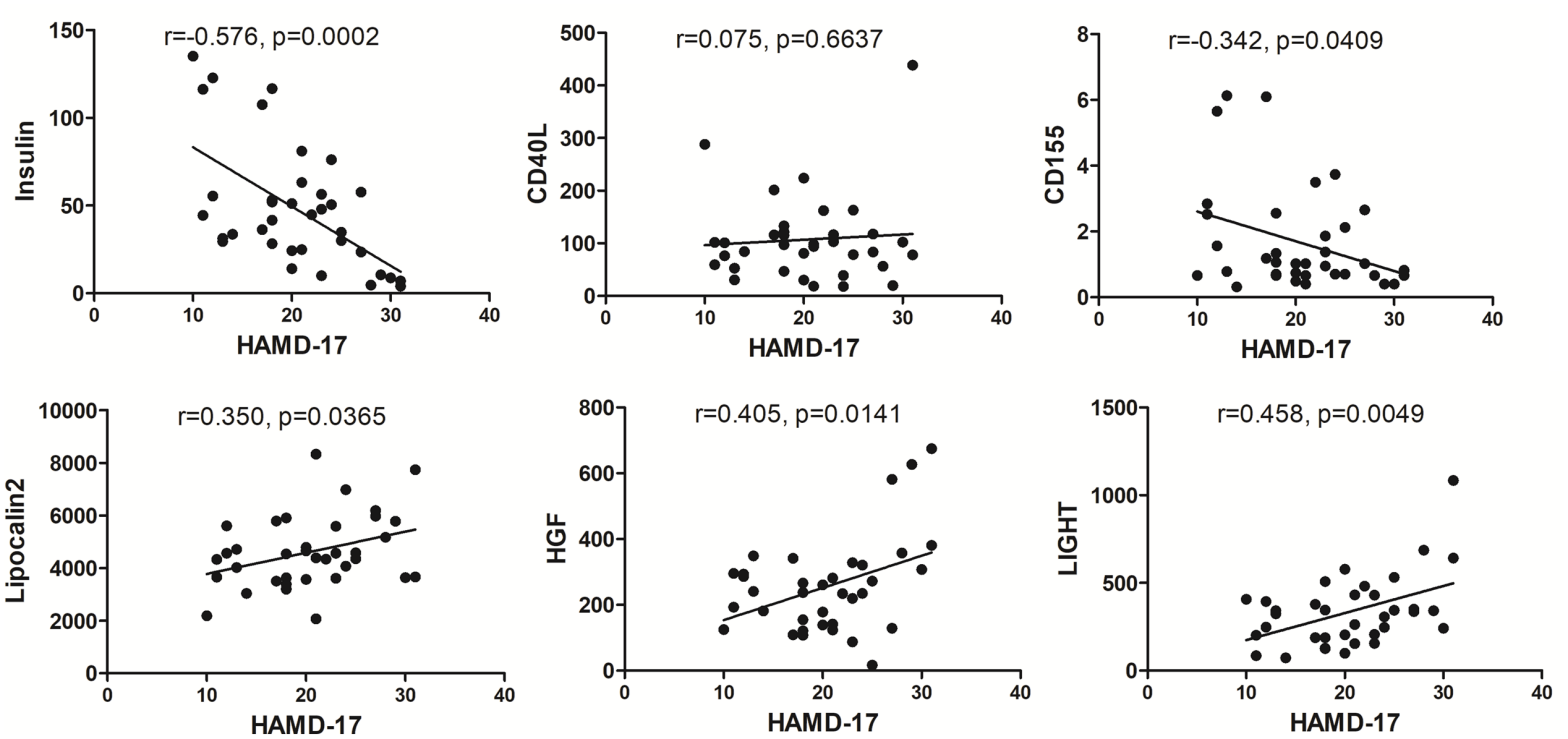
The correlation between each MDD-related characteristic proteins and HAMD-17. The levels of insulin, CD155, Lipocalin-2, HGF and LIGHT exhibited significant relationship with HAMD-17 scores in MDD group with *p* < 0.05.

Linear regression analysis between HAMD-17 scores and characteristic proteins showed that in MDD group there was negatively significant relationship between HAMD-17 scores and insulin, CD155, and there was positively significant relationship between HAMD-17 scores and Lipocalin-2, HGF and LIGHT (Figure 7).

## Discussion

Currently, except clinical symptomatologic criteria including ICD-10 or DSM-5, there is no specific test or biomarker for diagnosing and monitoring the progression of MDD. Furthermore, clinicians need at least 6 weeks to estimate the treatment effect of MDD, resulting in patients receiving inappropriate treatment. Therefore, a convenient, effective diagnostic tool is needed to reduce misdiagnosis and improve prognosis.

In this study, the antibody array technology for multiple cytokines measure showed 59 cytokines were significantly differential in MDD patients compared with healthy population including 21 down-regulated proteins and 38 up-regulated factors. The enrichment analysis showed some of these differential proteins are involved into the inflammatory response, and take part in the pathways including MAPK signaling, PI3K-Akt signaling, Ras signaling, NF-kappa B signaling, Rap1 signaling and JAK–STAT signaling which are known to activate the inflammatory response. In addition, cytokine-cytokine receptor interaction is also a mainly enriched pathway, and evidence showed that cytokines can permeate the blood–brain barrier by binding to cytokine receptors to induce inflammation (6). Taken together, these indicated that inflammation may play an important role in MDD. Previous studies revealed that inflammatory factors including IL-6, C-reactive protein, IFN-gamma, IL-10 and TNF-α were elevated in MDD patients (7–10), and the present study showed there were no significant differences in these inflammatory factors between MDD patients and healthy controls, suggesting the inflammatory mechanisms of MDD may be diversified. However, in these enriched pathways associated with inflammatory response, the differential proteins including insulin, SCF R, HGF, ANG1, Fas, IGF-1R, Flt-3 L, NT-4, VEGF R2, ErbB3, IL-2 Rb, CD40L, VCAM-1, LIGHT, MCP-4, MIP-1b, IL-9, IL-10 Rb, IL-21 and IL-28A were involved. Among these inflammatory factors, insulin, HGF, NT-4, VEGF R2, IL-2 Rb, CD40L, VCAM-1, MCP-4, MIP-1b and IL-9 were also found altered in MDD patients in previous studies (11–19), while SCF R, ANG1, Fas, IGF-1R, Flt-3 L, ErbB3, LIGHT, IL-10 Rb, IL-21 and IL-28A was firstly found to have abnormal levels in MDD patients as compared to healthy controls.

Furthermore, among these 59 differential proteins, LASSO regression was performed to obtain six characteristic proteins including three down-regulated proteins insulin, CD40L, CD155, and three up-regulated proteins Lipocalin-2, HGF and LIGHT. What is more, ROC curve analysis showed that the AUC values of these six characteristic proteins with more than 0.85 have a good property in discriminating MDD patients from healthy controls. Linear regression analysis showed a significant relationship between HAMD-17 scores and insulin, CD155, Lipocalin-2, HGF and LIGHT, indicating these proteins were independently associated with the severity of depressive symptoms in MDD patients.

Insulin is an endocrine peptide hormone that mediates glucose homeostasis. Insulin signaling is critical for neuroplasticity, cerebral metabolism, systemic energy metabolism (20, 21). In addition, insulin is also a potent neuroprotective agent, including inhibiting apoptosis, beta amyloid toxicity, oxidative stress and ischemia. Insulin facilitates learning and memory by modulating hippocampal synaptic plasticity. Although evidences showed that insulin resistance involved in the pathophysiology and treatment of mood and cognition disorders including MDD (22, 23), insulin is known to affect serotonin neurotransmission whose dysfunction will cause behavior and mood disorders, and it was reported that mood alterations during the postpartum period had an obvious decrease in circulating insulin levels (11). Likewise, the present study also showed that MDD patients had down-regulated circulating insulin levels compared with those in healthy controls. CD40 ligand (CD40L) is a transmembrane glycoprotein, which binds to the CD40 receptor and induces cytokines to upregulate the inflammatory response (24). Although some studies reported CD40L levels increased in patients with MDD compared with controls (25, 26), Myung et al. and our results showed that the level of CD40L in depressive patients was lower than that of healthy controls (16). Myung et al. thought this discrepancy was mainly due to the episode of depression. However, depressive patients in our study and those in previous studies with elevated CD40L levels were also conducted during the first episode of depression. Grewal IS et al. revealed that the CD40-CD40L interaction modulates the function of various effectors that serve as protective immune responses (27), and it was reported that the absence of CD40L resulted in rare chronic poliomyelitis and significantly aggravated the demyelination pathology in the CNS (28). Therefore, we speculated that down-regulated CD40L levels might reduce its protective immune responses to result in MDD. CD155 is a glycoprotein which interacts with several ligands such as CD226, TIGIT, CD96. The CD226/CD155 interaction regulates the pro-inflammatory (Th1/Th17)/anti-inflammatory (Th2) balance in humans (29). However, it had not revealed the association of CD155 with depression, and our study found circulating CD155 was reduced in MDD patients, indicating down-regulated CD155 might induce MDD by destroying the pro-inflammatory (Th1/Th17)/anti-inflammatory (Th2) balance. Lipocalin-2, as known as neutrophil gelatinase-associated lipocalin (NGAL), is a glycoprotein associated with a variety of inflammatory conditions (30, 31). Evidences have revealed that lipocalin-2 is an important neuro-inflammatory factor, and could reduce hippocampal neuronal growth during stress (32, 33). Naude et al. reported that increased circulating lipocalin-2 levels were associated with depression in patients with heart failure, as well as late-life depression (≥ 60 years) (34, 35). In the present study, we found circulating lipocalin-2 levels were also increased in younger patients with depression, indicating lipocalin-2 plays an important function in the pathophysiology of depression. Hepatocyte growth factor (HGF) is an angiogenic factor with the pleiotropic functions including the promotion of angiogenesis, cell survival, cell migration and anti-inflammation in a variety of cell types (36). HGF is also a key factor protecting neurons, preventing neuronal death, enhancing neuroregeneration. It was found that HGF was highly expressed at the nerve injury site for nerve repair (37). Moreover, previous studies found that HGF was elevated in women with postpartum depression (12), and our present research also found HGF was up-regulated in MDD patients. Over the decades, psychiatric researches have proved that inflammatory response is involved in the pathogenesis of MDD (38, 39). Therefore, we speculated that elevated HGF in depressive patients might play a self-protective function by inhibiting organic inflammatory response. LIGHT (TNFSF14) is a member of the tumor necrosis factor superfamily (TNFSF), which induces pro-inflammatory proteins expression via activating NF-κB (40). Although it was reported that altered TNFSF/TNFRSF expression was associated with brain-dependent behavioral and neuro-functional changes during neuroinflammation (41), LIGHT had not been found interrelated with depression. In the present study, circulating LIGHT levels were significantly higher in MDD patients, indicating LIGHT might participate in the pathogeny of depression by inducing other pro-inflammatory factors via activating NF-κB.

In conclusion, the present study revealed the levels of circulating 59 proteins altered in patients with MDD via a high-throughput antibody array technology, and the bioinformatics analysis showed theses differential proteins might involve in the pathogenesis of MDD via activating inflammation. Furthermore, among these 59 differential proteins, LASSO regression analysis found six characteristic proteins including insulin, CD40L, CD155, Lipocalin-2, HGF and LIGHT, which might be potential as diagnostic biomarkers in discriminating patients with MDD. In addition, insulin, CD155, Lipocalin-2, HGF and LIGHT might be useful to estimate the severity of depressive symptoms.

## Data availability statement

The original contributions presented in the study are included in the article/supplementary material, further inquiries can be directed to the corresponding author.

## Ethics statement

The study was approved by the ethics committee of The Fourth People’s Hospital of Wuhu City [(2020)-KY-18]. The patients/participants provided their written informed consent to participate in this study.

## Author contributions

EZ carried out all experiments and wrote the first draft of the paper. ZH and ZZ contributed to the sample collection and processing. XQ and JY conducted the statistical analysis. XS designed and revised the manuscript. All authors have read and approved the final manuscript.

## Funding

The research was supported by Key research and development plan of Anhui Province (Grant No. 202104j07020024), the Collegiate Major Natural Science Research Projects, Anhui Province, China (Grant No. 2022AH040178), and the Science and Technology Planning Project of Fourth People’s Hospital of Wuhu City (Grant No. kjxm2020-12).

## Conflict of interest

The authors declare that the research was conducted in the absence of any commercial or financial relationships that could be construed as a potential conflict of interest.

## Publisher’s note

All claims expressed in this article are solely those of the authors and do not necessarily represent those of their affiliated organizations, or those of the publisher, the editors and the reviewers. Any product that may be evaluated in this article, or claim that may be made by its manufacturer, is not guaranteed or endorsed by the publisher.
